# Dimorphism in *Neopseudocercosporella capsellae*, an Emerging Pathogen Causing White Leaf Spot Disease of Brassicas

**DOI:** 10.3389/fcimb.2021.678231

**Published:** 2021-06-04

**Authors:** Niroshini Gunasinghe, Martin J. Barbetti, Ming Pei You, Prabuddha Dehigaspitiya, Stephen Neate

**Affiliations:** ^1^ Centre for Crop Health, Institute for Agriculture and the Environment, Research and Innovation Division, University of Southern Queensland, Toowoomba, QLD, Australia; ^2^ School of Agriculture and Environment and the Institute of Agriculture, Faculty of Science, The University of Western Australia, Crawley, WA, Australia; ^3^ School of Agriculture, Food and Wine, Faculty of Sciences, University of Adelaide, Urrbrae, SA, Australia

**Keywords:** Arthroconidia, Blastoconidia, *Brassica*, dimorphism, morphological transformation, *N. capsellae*, white leaf spot

## Abstract

White leaf spot pathogen: *Neopseudocercosporella capsellae* causes significant damage to many economically important Brassicaceae crops, including oilseed rape through foliar, stem, and pod lesions under cool and wet conditions. A lack of information on critical aspects of the pathogen’s life cycle limits the development of effective control measures. The presence of single-celled spores along with multi-celled conidia on cotyledons inoculated with multi-celled conidia suggested that the multi-celled conidia were able to form single-celled spores on the host surface. This study was designed to demonstrate *N. capsellae* morphological plasticity, which allows the shift between a yeast-like single-celled phase and the multi-celled hyphal phase. Separate experiments were designed to illustrate the pathogen’s morphological transformation to single-celled yeast phase from multi-celled hyphae or multi-celled macroconidia *in-vitro* and *in-planta*. Results confirmed the ability of *N. capsellae* to switch between two morphologies (septate hyphae and single-celled yeast phase) on a range of artificial culture media (*in-vitro*) or *in-planta* on the host surface before infection occurs. The hyphae-to-yeast transformation occurred through the production of two morphologically distinguishable blastospore (blastoconidia) types (meso-blastospores and micro-blastospores), and arthrospores (arthroconidia).

## Introduction

Dimorphic fungi are capable of assuming two distinct morphologies during their life cycle, switching between a single-celled yeast form and a multi-celled hyphal form (Gauthier, 2015). Dimorphism is a highly synchronized, reversible response to an external stimulus posed by the environment (Lin et al., 2015). The majority of dimorphic fungi belong to the class Ascomycete or their ‘Imperfect’ relatives (Gauthier, 2015), and morphogenesis is widespread among pathogenic fungi of either mammals, plants, or insects (Gauthier, 2015). Well-known dimorphic phytopathogenic fungi such *as Taphrina deformans*, the cause of peach leaf curl disease, and *Ophiostoma novo-ulmi*, the cause of Dutch elm disease, have had major adverse impacts on agriculture or the urban landscape, respectively (Gauthier, 2015).

In dimorphic fungi, yeast phase development occurs usually in response to a change in growth condition and/or nutritional status of the growth medium (Odds and Kerridge, 1985; Lin et al., 2015). Although diverse stimuli, including pheromones, plant lipids, plant hydrophobicity, pH, nitrogen, and quorum sensing can induce the transition of yeast-to-mycelium or *vice versa* in fungal morphogenesis in plant pathogenic fungi (Orlowski, 1994), several human fungal pathogens are solely thermally dimorphic, where temperature alone induces the morphological transformation (Orlowski, 1994; Gauthier, 2015). The search for internal factors that regulate morphogenesis or external factors that stimulate this dimorphic switch is a rapidly growing area of research, due in part to the availability of novel microscopy and molecular tools in the past decade (Gauthier, 2015). Such studies have revealed that pathogenicity, virulence, and lifecycle of dimorphic fungal pathogens are closely interrelated, and that pathogenicity may be limited to one of the alternative morphologies (Gauthier, 2015; Lin et al., 2015).

*Neopseudocercosporella capsellae*, the cause of white leaf spot disease, causes significant damage to many economically important Brassicaceae crops, including oilseed rape/canola, vegetable, condiment, and fodder species (Barbetti and Sivasithamparam, 1981; Petrie, 1985; Cerkauskas et al., 1998; Gunasinghe et al., 2020). The pathogen produces foliar, stem, and pod lesions under favourable weather conditions (Petrie and Vanterpool, 1978) causing yield losses in the range of 24-30% in oilseed brassicas (Penaud, 1987; Barbetti and Khangura, 2000; Murtza et al., 2021). While the pathogen distribution is worldwide, it is most destructive in regions with cool and wet climates, such as occur in France (Perron and Nourani, 1990; Penaud and Walker, 2015), the United Kingdom (Inman et al., 1993), Canada (Petrie and Vanterpool, 1978), the United States of America (Ocamb et al., 2015) and Australia (Gunasinghe et al., 2016d; Van de Wouw et al., 2016; Gunasinghe et al., 2017; Murtza et al., 2018). Over recent decades, there has been an increase in white leaf spot disease in some countries, such as the United Kingdom following wetter/warmer winters in the 1990s (Inman et al., 1993).

Despite being a common *Brassica* pathogen, important knowledge gaps exist in the *N. capsellae*-*Brassica* pathosystem (Gunasinghe et al., 2020), particularly on critical aspects of its epidemiology. This is in part from its very slow growth rate on artificial media (Crossan, 1954) and its secretion of cercosporin, a non-host specific, photo-activated toxin into both artificial media and during the early infection process in host tissues (Gunasinghe et al., 2016b). Air-dispersed ascospores produced by the teleomorph are responsible for the disease initiation and long-distance pathogen dispersal in the United Kingdom (Inman et al., 1992). However, elsewhere, most *N. capsellae* populations are anamorphic and thought to be clonal, where aspects of *N. capsellae* pathogenicity, disease initiation, spread, and epidemic development are not fully described and/or understood (Gunasinghe et al., 2020). For example, on rapeseed cotyledons, Australian isolates of *N. capsellae* can be observed as hyphae, as large multi-celled conidia, and what appeared to be single-celled spores that are able to reproduce by budding (Gunasinghe unpub). This suggests that a morphologic shift between the yeast phase and the hyphal form likely occurs for *N. capsellae*. Its relevance is that morphologic shift between the yeast phase and the hyphal form in other pathogenic fungi is essential for maintaining pathogenicity (Orlowski, 1994). Further, Lin et al. (2015) noted, a thorough understanding of the life cycle of dimorphic pathogens is required if they are to be effectively controlled. Hence, studies were undertaken to determine if *N. capsellae* is dimorphic and able to switch between a single-celled yeast form and a multi-celled hyphal form *in-vitro* or *in-planta* on host leaves.

## Methods

### Pathogen Isolates

Three single-spored isolates of *N. capsellae* (UWA Wln-10, UWA Wlj-3, and UWA Wlra-7), stored as lyophilized ampoules containing the living pathogen preserved on vacuum dried agar cultures (Bazzigher and Kanzler, 1985) at room temperature, were obtained from the University of Western Australia (UWA) culture collection. All three isolates were collected within Western Australia. UWA Wln-10 was from infected leaves of *B. napus* at Bindoon North, UWA Wlj-3 was from diseased *B. juncea* leaves at Shenton Park, and UWA Wlra-7 was isolated from infected *Raphanus raphanistrum* leaves at West Calingiri (Gunasinghe et al., 2016d). Pathogen isolates were revived on Potato Dextrose Agar (PDA - Difco) or Malt Yeast Extract (MEA) plates and then working cultures were maintained as MEA media slants at 4°C. For *in-vitro* studies, two isolates of genetically different groups (UWA Wlj-3, and UWA Wlra-7) (Gunasinghe et al., 2016a) were used. When multi-celled conidia were needed, susceptible host plants grown in the field were spray inoculated with a mixture of hyphal fragments of all three isolates, and conidia produced on host plants lesions were collected (Gunasinghe et al., 2014).

### Preparation of Mycelial Suspension and Multi-Celled Conidial Suspension for *In-Vitro* and In-Planta Studies

To prepare a mycelial suspension, each of the three *N. capsellae* isolates (UWA Wln-10, UWA Wlj-3, and UWA Wlra-7) was sub-cultured onto MEA in Petri Plates and grown for three weeks at 20°C. Erlenmeyer flasks (250 mL) containing 150 mL of Malt Extract Broth (MEB) were inoculated with agar plugs from the growing edges of each *N. capsellae* culture separately and incubated on a rotary platform shaker (Innova™ 2100, New Brunswick Scientific) at 150 rpm and 20°C. After 3-4 weeks, cultures of all three isolates with abundant mycelial growth were mixed in equal volumes and blended for 5 min (Kambrook Mega Blender, Breville Group Ltd). Mycelial mats in the mixture after blending were removed by filtering through sterile gauze. Concentration of the filtered suspension of mycelial fragments containing 90 µm (± 10 µm) length was adjusted to 4 x 10^6^ mL^-1^ using a hemocytometer and sterile distilled water (Gunasinghe et al., 2014).

To produce multi-celled conidia, field-grown plants were inoculated with mycelial inoculum. Seeds of highly susceptible, *B. juncea* genotypes Rohini, Vardan, or Prakash (Gunasinghe et al., 2014) were sown (6 seeds per pot) in sequential batches of 9 pots each, seedlings were grown in a controlled environment room (15°C, 12h photoperiod and Photosynthetic Photon Flux Density of 580μmolm^-2^ s^-1^) for 15 to 20 days before transferring into an experimental field plot (1 x 0.5m) at the University of Western Australia, Crawley, Western Australia, where they were fertilized weekly with Thrive^®^ (DuluxGroup (Australia) Pty Ltd), a complete nutrient solution dissolved in water. Beginning at approximately four weeks of age, field plants were spray-inoculated weekly with a mixture of mycelial fragments (4 x 10^6^ fragments mL^-1^) prepared as described above for three consecutive weeks using a handheld and operated aerosol sprayer in the late afternoon to maximize the period of high humidity to favour infections.

After 20 to 25 days post-inoculation, when disease symptoms became apparent, leaves with typical white leaf spot symptoms were collected. Leaves with lesions were gently washed with sterile deionized water (DI) before, lesions were excised and placed into glass vials containing 20-30mL of sterile DI. Multi-celled conidia were released by vigorous handshaking for four min. The pieces of the leaf lesions were removed from the glass vials with a sterile pair of forceps and the mixture was centrifuged to pellet the conidia. The supernatant was discarded and the conidial pellet was washed twice in sterile DI water and re-suspended in 2 to 3mL of sterile DI water. Multi-celled conidial concentration (4 x 10^6^mL^-1^) was achieved by making adjustments based on hemocytometer counts (Gunasinghe et al., 2016c).

### Dimorphism *In-Vitro*

#### In*-Vitro* Blastospore Formation From *N. capsellae* Hyphae and Observations of Colony Morphology During Morphological Transformation

Morphological transformation of *N. capsellae* was examined by inoculating UWA Wlj-3, and UWA Wlra-7 on 5 different media: PDA (Difco), MEA (20g, peptone: 6g, dextrose: 20g, agar: 15g, de-ionized water 1L), V-8 juice agar (clarified V-8 juice: 200mL, CaC0_3_: 3g, agar 15g, de-ionized water 1L), Neutral Dox Yeast Agar (NDYA) (NaNO_3_: 2g, KH_2_PO_4_; 1g, MgSO_4_: 0.5g, KCL: 0.5g, FeSO_4_ 0.1% sol: 10mL, yeast extract: 0.5g, sucrose: 30g, agar: 14g, de-ionised water: 1L), Yeast Extract Peptone Dextrose Agar **(**YEDPA) (Yeast extract: 10g, peptone: 10g, dextrose: 20g, agar 15g, de-ionised water 1L adjusted to pH=6) or Water Agar (WA) (agar:15g, de-ionised water 1L) using two inoculation methods (streak plate method and agar plug method). A four week old pure mycelial colony of each isolate on NDY was used for all inoculations.

For the agar plug method, a mycelial plug (5mm diameter) from the growing edge of the mycelial colony was placed in the middle of a Petri Plate containing PDA, MEA, V-8 juice agar, NDYA, YEDPA, or WA. For the streak plate method, a loop full of mycelial fragments was taken from the growing edge of the colony and dragged over the surface of the plate in a ‘zig-zag’ formation. There were six single plate replicates for each medium for each inoculation method. Three plates from each media inoculated with each individual inoculation method were incubated at 23°C, and the other three at 37°C, in the dark for a maximum of 4 weeks.

Stereo microscopy, light microscopy (LM), and scanning electron microscopy (SEM) techniques were used to document colony and cell morphologies. Colony morphologies of *N. capsellae* on different media incubated at the two temperatures were recorded every other day by examining individual plates. Colonies on plates were further prepared to examine with a Nikon- SMZ1500 stereomicroscope, a Nikon Eclipse Ni-U light microscope, or SEM where necessary.

LM: Cells/hyphae collected by scraping the surface of colonies were mounted on microscope slides and stained with 1% cotton blue in lactophenol. Slides were studied and photographed (1000x magnification) with a Nikon Eclipse Ni-U light microscope. SEM: Selected colonies were prepared for SEM for further analysis. Thin agar blocks (2-3 cm) with colonies were cut carefully from the culture plate with a sterile scalpel and placed on a pin mount with a carbon tab (ProSci Tech) on it or in cryovials (2mL). The samples on pin mounts were air-dried in a laminar flow cabinet for five days. Cryotubes with samples were frozen at -80°C (Revco ULT390-5-D, Thermo Scientific) for 24h and then freeze-dried (FD-8 Witeg Germany) for 24h. Dried samples were carefully placed on standard pin mounts with a carbon tab on top. Finally, all samples were sputter-coated with Mini Plasma Sputter Coater (gold: 0.1mm) and visualized and imaged using a Jeol JCM-6000 Versatile Benchtop SEM.

#### *In-Vitro* Blastospore Formation From *N. capsellae* Multi-Celled Conidia

Two drops (20µl) from multi-celled conidial suspension prepared as described before in the previous section on preparation of multi-celled conidial suspension, were placed on 10 standard glass microscope slides and incubated in a moist chamber. After a 24h incubation period, 1% cotton blue in lactophenol was added to each drop, before examining under an Olympus BX5 microscope. From 5 random slides, a total of 50 fields (10 from each) were imaged with an Olympus DP71 digital photographic system.

#### Morphological Analysis of *In-Vitro* Formed Blastospores (Single Spore/Cell) Types

Two measurements (length and width) were taken from more than 400 spores using the software ImageJ 1.53a (Schneider et al., 2012) from digital images taken of two different single-celled blastospore types (micro-blastospores and meso-blastospores) produced from *N. capsellae* hyphae in pure cultures or multi-celled macroconidia suspended in sterile distilled water. All the measurements for smaller bacteria-like spores (micro-blastospores) were taken from SEM images, and the larger yeast-like spore (meso-blastospore) measurements were taken from a combination of both SEM and LM images.

### Dimorphism *In-Planta*

#### Host Genotypes

Two genotypes from each of three major oilseed species were selected for *in-planta* studies. The six susceptible or resistant genotypes used were *B. carinata* (ATC94129P, highly resistant and UWA#012, very susceptible)*, B. napus* (Hyola 42, resistant and Trilogy, susceptible), and *B. juncea* (Dune, moderately resistant and Vardan, susceptible) (Gunasinghe et al., 2014).

#### *In-Planta* Blastospore Formation From *N. capsellae* Hyphae

Seeds of each genotype were sown in 48 cell seedling trays (30 x 34 cm), four seeds per cell and four replicate cells per genotype. Ten days after sowing, plants were thinned to two per cell. Seedlings were grown in a controlled environment room (15°C, 12h photoperiod, and Photosynthetic Photon Flux Density of 580 μmol m^-2^ s^-1^). Ten-day-old cotyledons of each seedling were inoculated by depositing a single drop (10µl) of the mycelial suspension (4 x 10^6^ mL^-1^), prepared as described earlier in the previous section on preparing of mycelial suspension, on each cotyledon lobe. The control was an identical tray of seedlings under the same conditions but inoculated with sterile distilled water. All plants were placed in clear polyethylene bags for 48h to maintain high humidity to promote infection (Brun and Tribodet, 1991). Five cotyledons for each genotype, (4 inoculated and 1 control from each of the 5 cells) were randomly sampled for LM or SEM 48h post-inoculation.

#### *In-Planta* Blastospore Formation From *N. capsellae* Multi-Celled Conidia

Collection of multi-celled macroconidia from diseased leaves and preparation of macroconidial suspension in sterile distilled water (4 x 10^6^ mL^-1^) was done as described in the previous section on preparation of muiti-celled conidia. An experiment identical to mycelial inoculation studies was conducted, but by inoculating cotyledons with a 10µm multi-celled conidial suspension rather than mycelial suspension. After 48h post-incubation, two sets of five cotyledons (four inoculated and one control) were collected separately for LM and SEM.

#### Sample Preparation for Microscopy and Morphological Analysis of Blastospores (Single Spore/Cell Types) Produced *In-Planta*

For LM studies, collected cotyledons were decolourized with acetic acid: ethanol: water (2:2:1) solution and stained with 1% cotton blue in lactophenol for 40 sec to prepare whole wet mounts (Gunasinghe et al., 2016c). Four inoculated and one uninoculated cotyledons were examined using an Olympus (BX51) microscope, where 25 random images from each inoculated site (50 from a cotyledon and a total of 50 x 4 = 200 for each genotype) were captured using an Olympus DP71 digital photographic system.

For SEM studies, cotyledon samples were fixed in 2.5% glutaraldehyde and cut into 4 x 4 mm pieces and transferred into glass vials with fresh 2.5% glutaraldehyde. Samples were then prepared for SEM using a BioWave microwave processor fitted with a PELCO coldspot (Gunasinghe et al., 2016c). Prepared samples were mounted on standard aluminium pin mounts with carbon tabs (ProSciTech), sputter-coated with 5 nm carbon, 3 nm platinum, and surface imaged with a field emission SEM (Zeiss Supra 55 VP). Twenty-five random images were taken from each of the inoculated areas (50 from a cotyledon and a total of 50x4 = 200 for each genotype).

Different blastospore types formed from *N. capsellae* multi-celled macroconidia or hyphal fragments were measured using the software ImageJ 1.53a. Two measurements from more than 400 micro-blastospores (bacteria-like single-celled spores) from SEM images and meso-blastospores (Yeast-like single-celled spores) from a combination of both SEM and LM images were taken.

### Molecular Identification

Confirmation of the identity of the two observed morphologically different phases (Hyphae and single-celled blastospores) was undertaken by sequencing the internal transcribed spacer region (ITS1, 5.8S, and ITS4) of the rDNA operon, and comparing sequence data with available sequence information data for *N. capsellae* isolates in GenBank. Areal hyphae obtained from a pure mycelial colony and single-celled blastospores collected from a young bacterial-like colony consisted only of blastospores have been used to extract DNA. To obtain the hyphal phase, five Petri Plates containing 15mL NDY were inoculated with a mycelial plug taken from a three-week-old culture of the isolate UWA Wlj-3 on MEA plates at the mycelial phase. After 20 to 30 days of incubation at 23°C, plates were examined to select a plate with mycelial growth and no blastospores. Aerial mycelium (hyphae) from selected NDY cultures was harvested and placed in a sterile Eppendorf tube. Streak plates of YEPD (in duplicate) from the same isolate: UWA Wlj-3 were prepared from the same mycelial colony on MEA to obtain yeast phase (single cells). YEDP was used to induce the unicellular phase of the pathogen (Zhu et al., 2017). After two days of incubation at 23°C in dark, cells from the growing yeast-like colonies were aseptically transferred into a sterile Eppendorf tube.

#### Extraction of Genomic DNA, PCR, and Sequencing

Genomic DNA was extracted using the ISOLATE II Genomic DNA Kit (Bioline (Aust) Pty Ltd) with some modifications to the standard protocol. All samples with mycelia or single cells (yeast phase) were washed with 400µl of TE buffer in an Eppendorf tube, pelleted by centrifugation (13,000rpm, 5min) in a microfuge^®^ 18 (Beckman Coulter ™) and the supernatant was discarded. Lysis buffer (200µl) and two metal beads were added to each of the sample and homogenized with the tissue homogenizer (FastPrep-24TM 5G Homogenizer – MP Biomedicals) for 6 seconds. Genomic DNA extraction in the homogenized samples was carried out using the ISOLATE II Genomic DNA Kit according to manufacturer instructions. All DNA samples were stored at 4°C after evaluating the quality and concentration of DNA extracted using a DeNovix: DS-11 FX Spectrophotometer/Fluorometer.

Part of the nuclear rRNA operon was amplified with primers ITS1 (5’TTT CCG TAG GTG AAC CTG C3’) and ITS4 (5’TCC TCC GCT TAT TGA TAT GC3’) using the polymerase chain reaction (PCR) (Gunasinghe et al., 2016a). Spacer I, between the 18S and 5.8S rRNA gene, was amplified using the 5’ primer ITS1, and spacer II, between 5.8S and 28S of the rRNA gene, was amplified with the 3’ primer ITS4. PCR reactions were carried out with 50ng genomic DNA in MyTaq Red mix (Bioline (Aust) Pty Ltd). PCR conditions were: initial denaturation step at 94°C for 2 minutes, followed by 35 cycles of 1 minute at 94°C, annealing at 55°C and extension for 1.5 minutes at 72°C and a final elongation step of 72°C for 2 min (Gunasinghe et al., 2016a). PCR products were separated and visualized by gel electrophoresis at 84V for 45 minutes on a 1% (w/v) agarose gel and products of 500bp (20µl of each) were sequenced by Macrogen Inc. (Korea).

## Results and Discussion

**Figure 1:**
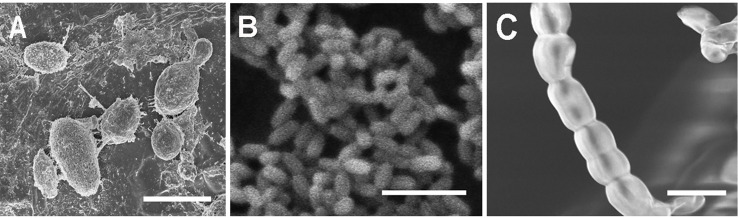
Scanning electron micrographs of single-celled spore types with different morphologies produced by *Neopseudocercosporella capsellae* during morphological transformation from hyphae-to-yeast. **(A)** Unicellular meso-blastospores. **(B)** Unicellular micro-blastospores. **(C)** Arthrospore chain. Scale bars = 5µm.

Observations confirmed the morphogenic plasticity of *N. capsellae*. The species changed its morphology from septate hyphal growth to budding yeast form or *vice-versa* depending on external conditions. The morphological transformation (hyphae-to-yeast) of *N. capsellae* resulted in two common single-celled blastospore (blastoconidia) types distinguished based on gross appearance: The first, predominantly elliptical and comparatively large single-celled meso-blastospores that resembled yeast cells where average dimensions were 4.8 x 2.7µm (**Table 1** and **Figure 1A**) and the second, single-celled micro-blastospores (average dimensions of 1.9 X 0.9 µm) that resembled rod-shaped bacterial cells (**Table 1** and **Figure 1B**) and comparatively large, spherical to cylindrical arthrospores (arthroconidia) (**Figure 1C**). The formation of blastospores (Odds and Kerridge, 1985; Gauthier, 2015; Lin et al., 2015) and arthrospores (Barrera and Szaniszlo, 1985) are important aspects of fungal morphogenesis in many dimorphic or polymorphic fungal species, including Ascomycetes. Blastospores are asexually produced single-celled spores that can increase in number rapidly through repeated budding (Odds and; Kerridge, 1985; Mehrabi et al., 2006; Lin et al., 2015), while arthrospores are formed by segmentation and subsequent fragmentation of existing hyphae (Barrera and Szaniszlo, 1985).

**Table 1:** Spore dimensions (µm) of two types of blastospores: meso-blastospores and micro-blastospores.

	Meso blastospore *in-planta*	Meso blastospore *in-vitro*	Micro blastospore *in-planta*	Micro blastospore *in-vitro*
Average Width	2.9 ± 0.82	2.7 ± 0.64	0.65 ± 0.15	0.9 ± 0.34
Range	0.9-6.6	1.0-4.9	0.2-1.0	0.3-2.0
Average Length	3.4 ± 0.95	4.8 ± 1.24	1.1 ± 0.13	1.9 ± 0.44
Range	0.9-7.1	2.3-9.2	0.3-2.5	0.7-3.4

Values are the mean of more than 400 cells from each morphotype measured *in-vitro* and in-planta.

This morphological transition in *N. capsellae* has not been previously recognized. To eliminate the potential for contamination being the source of the newly described structures, the identity of the structures was confirmed by PCR amplification and sequencing of the ITS1 and ITS4 regions of nuclear ribosomal DNA of blastospores taken from a yeast-like colony (GenBank Acc. No. MZ149251) and hyphae from a pure mycelial colony (GenBank Acc. No. MW898135). The BLAST results for both samples were 99% match to *N. capsellae*.

### *In-Vitro* Morphogenesis

#### Pure Cultures on Growth Media

**Figure 2:**
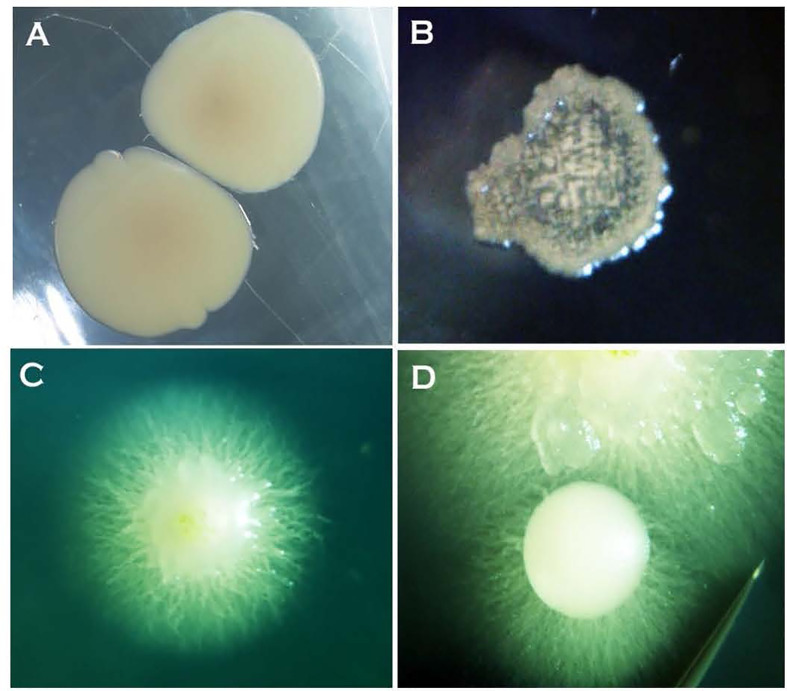
Two-day-old bacteria-like colonies of *Neopseudocercosporella capsellae* consisting of single-celled blastopores appeared after streak-inoculation using hyphal fragments from a pure hyphal colony. **(A)** Isolate UWA Wlj-3 on NDY and **(B)** isolate UWA Wlra-7 on PDA. **(C)** and **(D)** Ten-day-old colonies of isolate UWA Wlj-3 on PDA where hyphae initiation is evident at the periphery at the transition stage from a bacterial-like initial stage.

**Figure 3:**
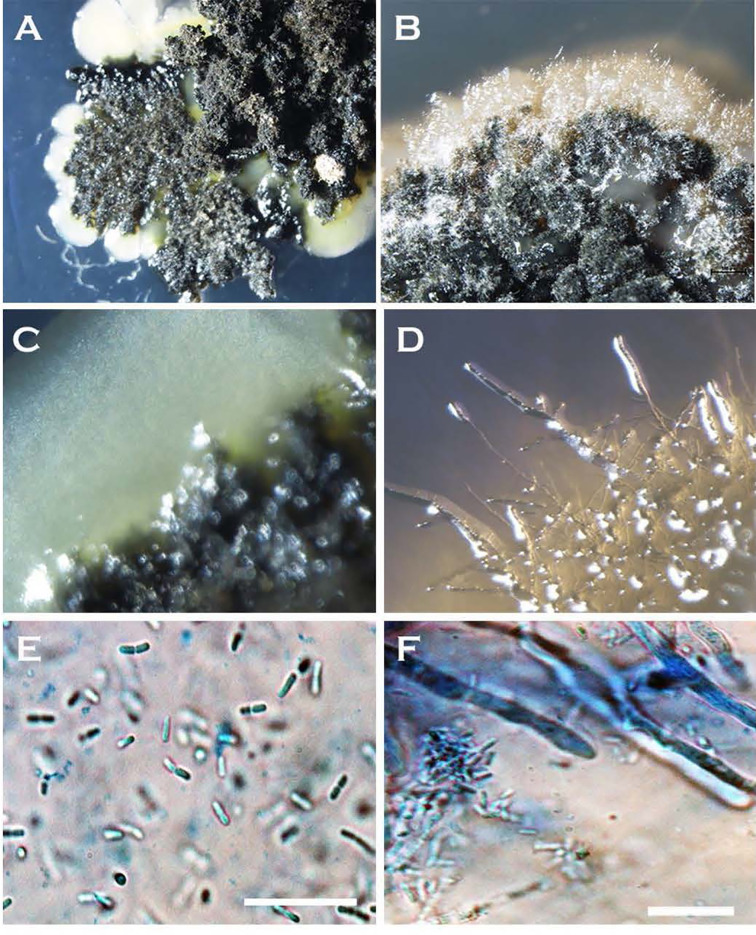
Morphological transformation (hyphae-to-yeast) of *Neopseudocercosporella capsellae* cultures on growth media where blastospores are seen as creamy white to tan masses at the edges of colonies **(A–D)**. Colonies at the time of hyphae to yeast transformation, **(A)** seven week old colony of isolate UWA Wlj-3 on NDY, and **(B)** five week old colony of isolate UWA Wlra-7 on PDA. **(C)** Edge of the UWA Wlj-3 colony, and **(D)** edge of UWA Wlra-7 colony, where hyphae-to-yeast transformation occurred. **(E)** Blastospores from UWA Wlj-3 colony and **(F)** hyphae from UWA Wlra-7 colony, both stained with 1% cotton blue in lactophenol and as seen under the light microscope. Scale bar = 5µm.

The formation of single-celled spores (blastospores and arthrospores) produced prominent changes in colony morphology (**Figures 2** and **3**). Morphological transformation from hyphae-to-yeast or yeast-to-hyphae was observed on all 5 tested media, although some different media favoured the formation of yeast and the other the formation of mycelial phase. Transferring hyphae to a fresh medium (subculturing) initiate yeast-like colonies on some media demonstrating hyphae-to-yeast transformation. Generally, on commonly used media like PDA or NDY, and WA, the mycelial phase dominated with an infrequent, short, initial yeast-like phase immediately after subculturing. When yeast-like growth was observed initially (**Figures 2A, B** **)**, yeast-to-hyphae transition was initiated in colonies anytime within two-three weeks (**Figures 2C, D** **)**. The hyphae-to-yeast transformation was also observed in mycelial colonies older than one month (**Figure 3**). At the initial growth stage on some media such as NDY, PDA, and WA, the yeast phase was present but visible only under the microscope and the proportion of yeast to mycelia and the time taken for the yeast-to-hyphae transformation differed across the media tested.

Subculturing and the method of inoculation influenced the morphological transformation of the fungus. Placing *N. capsellae* hyphal fragments onto a new growth medium promoted the initial formation of creamy white-to-tan-coloured colonies dominated by single-celled blastospores (**Figures 2A, B** **)**. Similar growth behaviour on agar media by dimorphic pathogens has been observed. Initially formed yeast-like colonies of *Candida citri* switched to mycelial growth after prolonged incubation (Sipiczki, 2011). Odds and Kerridge (1985) claim that the *C. albicans* blastospore-dominating yeast phase was easy to obtain and maintain on artificial media, while the pure mycelial phase was difficult to attain as it is only a transient form.

**Figure 4:**
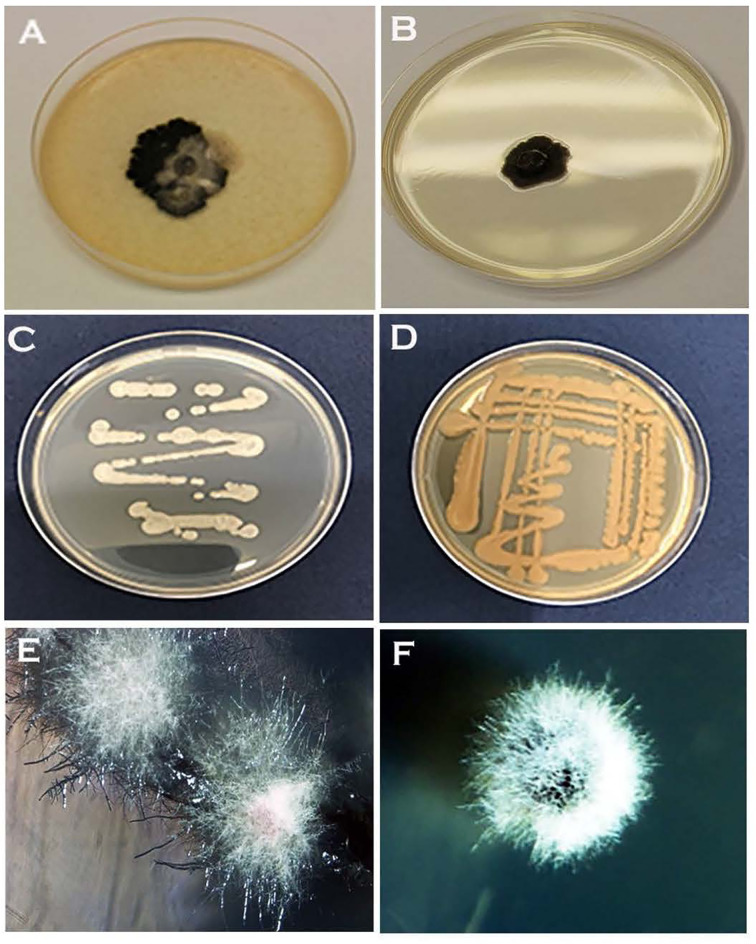
Different colony morphologies of *Neopseudocercosporella capsellae* isolate UWA Wlj-3 grown on three different growth media, and with two different subculturing methods using a hyphal colony on a single growth medium. **(A)** Seven week old colonies on V8 juice agar and **(B)** PDA, inoculated with an agar plug. Four-day-old streak plate on **(C)** PDA and **(D)** V8 juice agar. **(E)** Four-week-old hyphal colonies on WA streak plate. **(F)** Three-week-old hyphal colony on NDY inoculated with an agar plug.

Of the two inoculation methods tested, streaking favoured the formation of the yeast phase. Hyphal growth with or without initial yeast-like growth was observable on PDA, NDY, and V8 juice agar when inoculated with an agar plug (**Figures 4A, B, F**). Except for WA, yeast-like colonies appeared on streak plates of all media within 24h of inoculation (**Figures 4C, D** **)**. On WA, yeast-like colonies, hyphal growth, or both were observed during the initial growth stage depending on the inoculation method used (**Figure 4E**). The appearance of initial yeast-like colonies immediately after subculturing was fast, within overnight incubation (**Figures 4C, D** **)**, while it took at least 10-14 days for hyphal growth to occur when the initial yeast growth was absent (**Figures 4E, F**).

**Figure 5:**
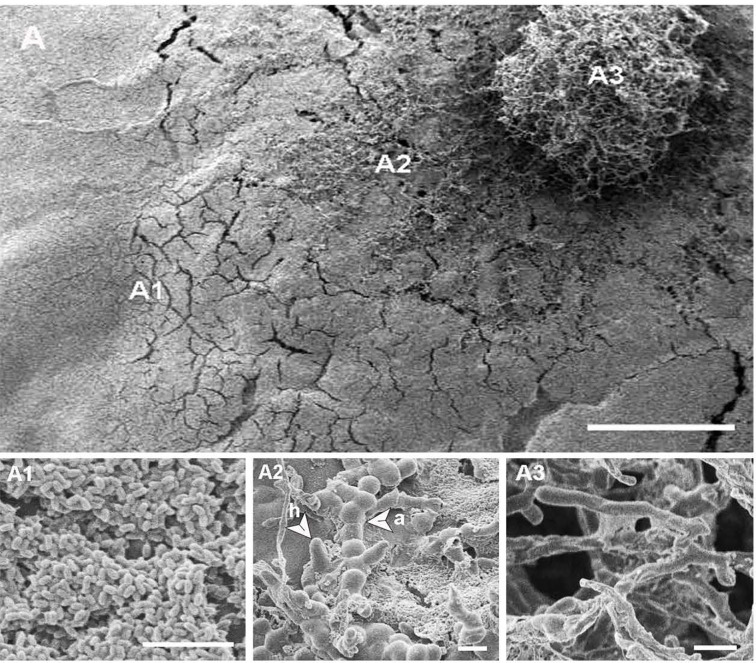
**(A)** Scanning electron micrograph of a three-week-old heterogeneous *Neopseudocercosporella capsellae* isolate UWA Wlj-3 colony on PDA, showing cell types with different morphologies at different places in the culture, scale bar = 500µm. **(A1)** Blastospores (yeast cells) dominating at the periphery of the colony. **(A2)** Middle area of colony showing a mixture of blastospores, arthrospores (a) and hyphae (h). **(A3)** Centre of the colony dominated by true hyphae. Scale bars = 10µm.

The co-occurrence of cells with different morphologies varying from single-celled blastospore types to true hyphae in a single colony was observed in *N. capsellae* colonies (**Figure 5**). However, the dominating morphotype and the architecture of the colonies seem to depend on several factors, such as isolate, method of inoculation, and type of the growth medium and changed over time with the age of the colony (**Figures 2** and **3**). The formation of mixed colonies with different cell morphologies has been reported in other dimorphic pathogens such as *Cryptococcus* (Levy et al., 2012; Lin et al., 2015) and *Candida* sp. (Odds and Kerridge, 1985; Sipiczki, 2011). *Cryptococcus* produces colonies with different morphologies, where yeast cells (blastospores) predominate in the colony centre and hyphae dominate at the periphery (Lin et al., 2015). Obtaining a colony consist of single-cell morphology was a difficult task for *C. albicans* on artificial culture media because of the co-existence of blastospores, hyphae, and pseudohyphae (Odds and Kerridge, 1985). Such colonies, the architecture of the colony, degree of morphological heterogeneity, and the proportion of each cell morphology of otherwise genetically identical cells, are a result of differential gene expression among cells (Levy et al., 2012) as influenced by one or more of several factors, such as gradient of signalling molecules created by the varying microenvironments across the growth medium and/or factors like the age or cell-cycle stage (Lin et al., 2015).

Apart from much larger colonies at 37°C, the same growth patterns were observed at both temperatures (23°C and 37°C). Therefore, these *in-vitro* studies at two temperatures suggest that *N. capsellae* morphogenesis is not induced solely by temperature as seen in thermally dimorphic human fungal pathogens (Dukik et al., 2017), but by more complex external and internal factors, including the growth medium.

#### Multi-Celled Macroconidia in Sterile Distilled Water

**Figure 6:**
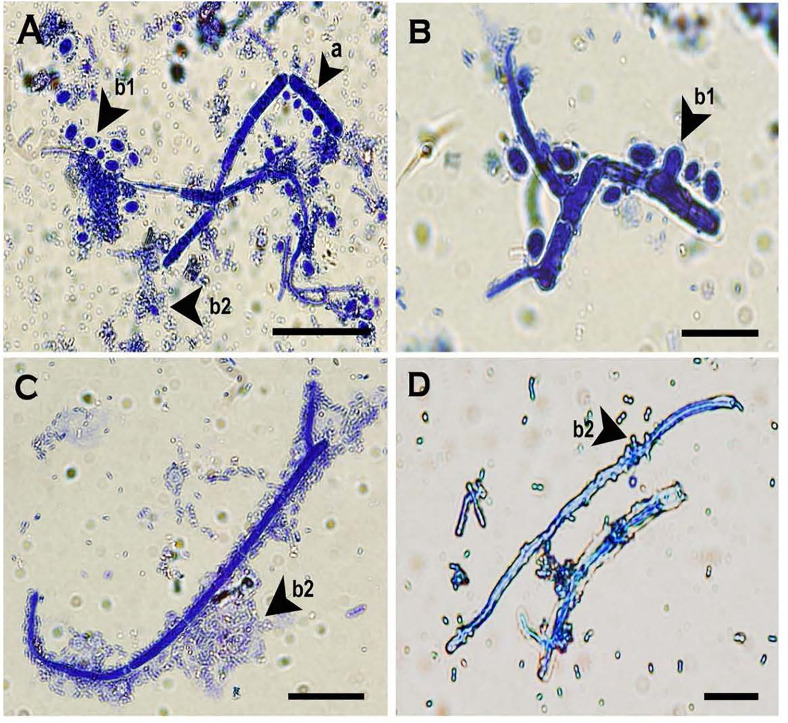
Two morphologies of blastospores and arthrospores formed from *Neopseudocercosporella capsellae* multi-celled macroconidia suspended in sterile distilled water 24h post-inoculation. **(A)** Multi-celled macroconidia with meso-blastopores (b1), micro-blastospores (b2) and an arthrospore (a). **(B)** Multi-celled macroconidium producing meso-blastospores (b1) (arrow shows a meso-blastospore bud raised adjacent to the septa). **(C)** Multi-celled macroconidium with micro- blastospores (b2). **(D)** Micro-blastospore (b2) formed from multi-celled macroconidia (arrow shows buds of micro-blastospores). Scale bars **(A, C)** 20µm; **(B, D)** 10µm.

Multi-celled macroconidia produced both micro-blastospores and meso-blastospores or arthrospores *in-vitro* within a 24h incubation period. All three spore types were stained with cotton blue and observable under LM among the multi-celled macroconidia when suspended in sterile distilled water (**Figure 6**). However, not all multi-celled conidia produced single-celled blastospores, as some germinated by producing single or multiple germ tubes.

### *In-Planta* Morphogenesis

**Figure 7:**
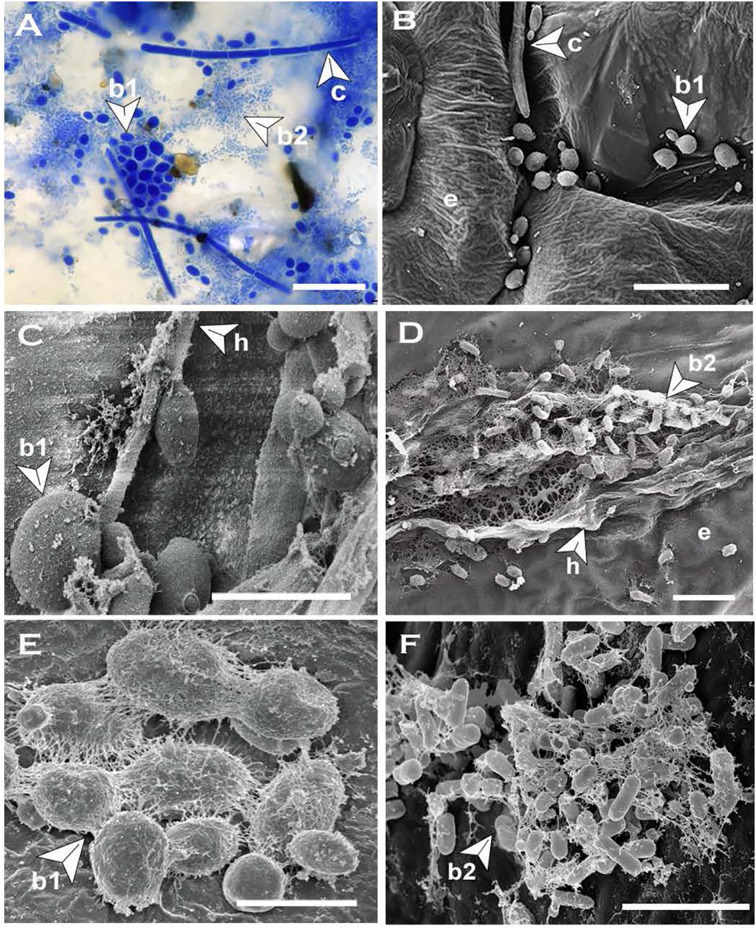
Blastospores produced by *Neopseudocercosporella capsellae* on cotyledons of the host Brassica juncea or *B. napus.* **(A)** Multi-celled macroconidia (c), meso-blastospores (b1) and micro-blastospores (b2) on a cotyledon surface 48h post-inoculation with a multi-celled macroconidial suspension (cotyledons were decolorized by immersing in acetic acid:ethanol:water (2:2:1) solution at 25°C for 3-4 days before visualization under the light microscope (scale bar= 20µm). **(B)** Cotyledon surface inoculated with a multi-celled macroconidial suspension showing multi-celled macroconidia (c), meso-blastospores (b1), and epidermal cells (e) as seen under Scanning Electron Microscope (SEM) (Scale bar=10µm). **(C, D)** Cotyledon surface inoculated with hyphal fragments showing hyphae (h) producing meso-blastospores **(C)** (Scale bar=5µm) and micro-blastospores **(D)** (Scale bar=1µm) as seen under SEM. **(E, F) ** Morphology of meso-blastospores **(E)** (Scale bar=5µm) and micro-blastospores **(F)** (Scale bar=1µm) produced in planta as seen under SEM.

Multi-celled macroconidia or hyphal fragments newly introduced onto host tissue (cotyledons) formed single-celled blastospores or arthrospores displaying the same trend of morphological transition that occurred *in-vitro*. All single-celled spore types similar to the spore types produced *in-vitro* (meso and micro-blastospores and arthrospores) were evident on cotyledons inoculated with multi-celled macroconidia suspensions (**Table 1** and **Figures 7A, B, E, F**) or mycelial fragments (**Figures 7C, D** **)** within 48h post-inoculation and this was consistent across all six test genotypes belonging to three different host species. However, germinated multi-celled macroconidia with single or multiple germ tubes were also common. Initial observations were that the amount of germinating multi-celled macroconidia or each morphologically different single-celled blastospore type could vary depending on the host, such as susceptible and resistant host species. After landing on the host tissue, the formation of a single-celled blastospore (yeast phase) on the host surface just before invasion has been observed for several dimorphic plant pathogens (Ingold, 1995; Ruiz-Herrera et al., 1995; Vollmeister et al., 2012; Cissé et al., 2013). Asexually produced diploid, multi-celled pycnidiospores of *Zymoseptoria tritici*, a dimorphic wheat pathogen causing Septoria tritici blotch (Palmer and Skinner, 2002; Mehrabi et al., 2006) or teliospores of *Ustilago maydis*, a maize pathogen causing smut disease (Ruiz-Herrera et al., 1995; Vollmeister et al., 2012), establish a short yeast-like phase on the leaf surface by producing blastospores before host invasion in the filamentous form. In *Taphrina deformans*, the cause of leaf curl disease of peach and nectarine, ascospores discharged from curled leaves produce single-celled blastospores, that overwinter and then switch back to the infectious filamentous form to invade the host (Rodrigues and Fonseca, 2003; Tavares et al., 2004; Cissé et al., 2013). This morphological transition from multi-celled conidia or hyphae-to-yeast and yeast-to-hyphae plays unique species-specific roles in virulence (Mehrabi et al., 2006; Gauthier, 2015; Lin et al., 2015). For instance, yeast cells of *T. deformans* function as an overwintering dormant spore (Cissé et al., 2013), while dissemination is the key role of yeast cells produced by *Ophiostoma ulmi* (Lin et al., 2015).

### Blastospore (Blastoconidia) Formation

**Figure 8:**
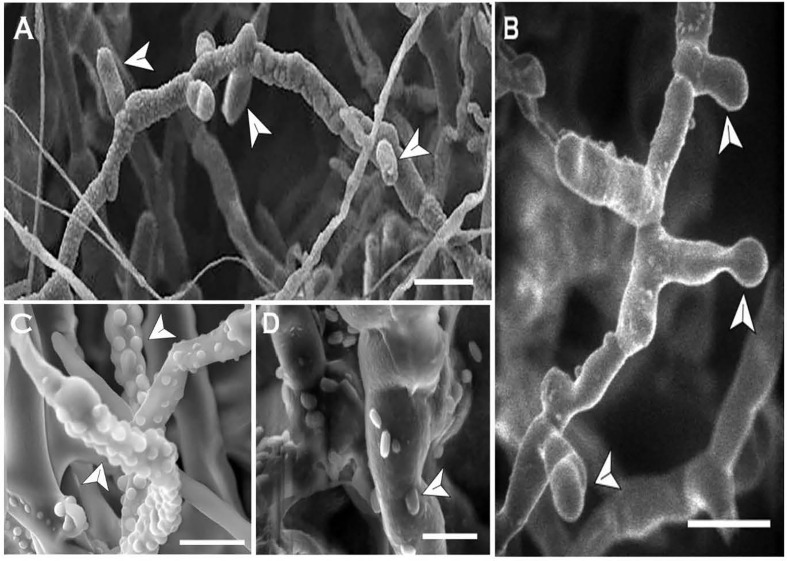
*In-vitro * blastospore formation by *Neopseudocercosporella capsellae* hyphae as seen under the scanning electron microscope. Old septate hyphae producing meso-blastospores (arrows) at the septa **(A)** and the tip **(B)** at the transition stage (Scale bars =10µm). **(C)** Young hyphae showing buds for micro-blastospores (Scale bar = 5µm). **(D)** Old hyphae with micro-blastospore buds and single-celled micro-blastospores (arrows) separated from the original hyphae (Scale bar = 2µm).

In *N. capsellae*, blastospores are produced during hyphae-to-yeast transition from multi septate hyphae (**Figure 3**) or by asexually produced multi-celled macroconidia (**Figures 6** and **7**). Although there can be a variety of different morphologies observed, the formation of blastospores involves three steps of bud emergence, bud growth, and spore/conidia separation. The sites at which blastospores are produced during hyphae-to-yeast transformation could be limited and for *C. albicans* these hyphal sites are adjacent and posterior to septa (Odds and Kerridge, 1985). Similarly, in *N. capsellae* yeast-like meso-blastospore formation occurred at the septa of hyphae (**Figures 8A, B** **)** or the tip (**Figure 8B**). However, micro-blastospore formation from hyphae was random and occurred in comparatively large numbers (**Figures 8C, D** **)**.

### Arthrospore (Arthroconidia) Formation

The formation of single-celled arthrospores from existing hyphae has long been observed during fungal morphogenesis in certain fungal species (Miyazi and Nishimura, 1971; Bibel et al., 1977; Emyanitoff and Hashimoto, 1979; Cartwright et al., 2014). These single-celled spores are produced in extensive numbers to enhance the pathogen’s reproductive potential and, therefore, the primary function of arthrospore is reproduction rather than survival between seasons. However, arthrospores can also be the principal means of dissemination in certain pathogenic fungi (Barrera and Szaniszlo, 1985). Arthroconidia formation and morphogenesis in dimorphic human fungal pathogens can be stimulated by several types of environmental stress conditions related to growth medium composition, carbon dioxide tension, and increased temperatures (Rippon and Scherr, 1959; King and Jong, 1976; Bibel et al., 1977; Emyanitoff and Hashimoto, 1979). Initiation of arthro sporogenisis, the age of conidia formation, and the number of conidia formed are all highly species-specific (Barrera and Szaniszlo, 1985).

**Figure 9:**
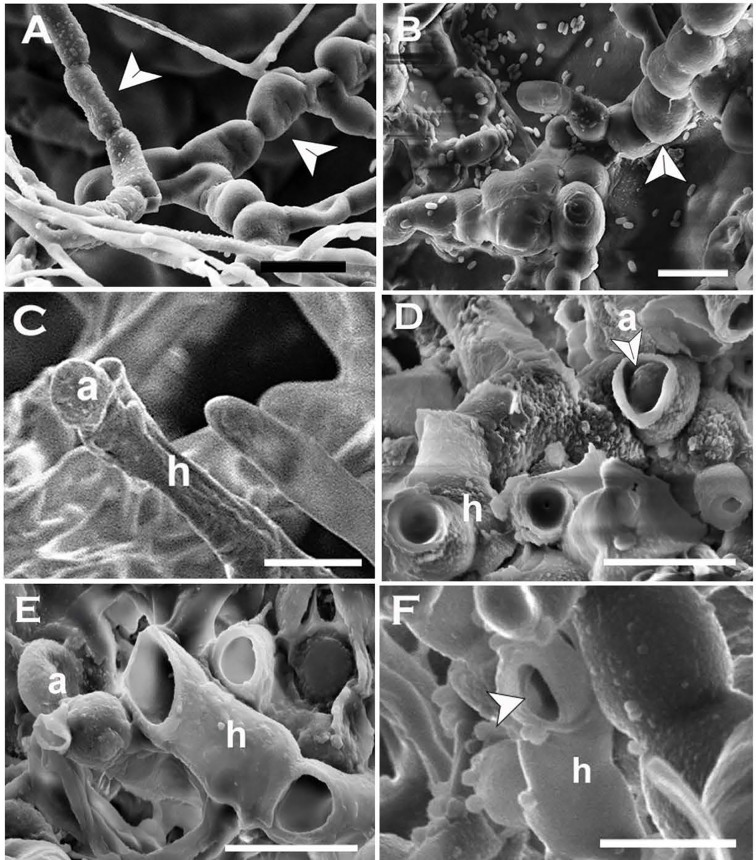
Formation and release of arthrospores (arthroconidia) produced *in-vitro* from *Neopseudocercosporella capsellae* hyphae as seen under the scanning electron microscope. **(A, B)** Series of delicately attached arthrospores (arrows) produced by 7 week old colonies of isolate UWA Wlj-3 on NDY **(A)** and PDA **(B)**. The arthrospores break off and disseminate when disturbed (Scale bars = 5µm). **(C, D)** Releasing spherical arthrospore (a) after transverse dehiscence of the hyphae (h) at the septa. **(D, F) ** Hyphal cell walls after releasing arthrospores by dehiscence along the septa **(C)** or centripetal dehiscence **(D) **(Scale bars = 5µm).

**Figure 10:**
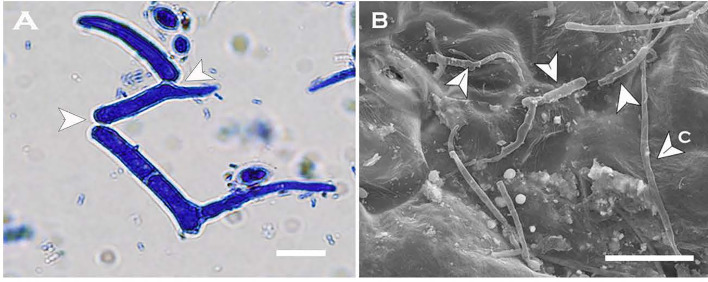
Arthrospores produced by *Neopseudocercosporella capsellae* multi-celled macroconidia on the host. **(A)** Cylindrical arthrospore formation by disintegration of original macroconidia cell walls at septa (arrows) on the cotyledon surface as seen under the light microscope. **(B)** Scanning electron micrograph of multi-celled macroconidia (c) and germinating three arthrospores as single-celled spores by producing a single germ tube each (arrows) on the cotyledon. Scale bars = 10µm.

Arthro sporogenesis of *N. capsellae* was observed *in-vitro* from multi-celled hyphae on different artificial media (**Figure 9**) or multi-celled macroconidia (**Figure 10**). This is the first report showing arthrospores from *N. capsellae*, although the cleavage of multi-celled macroconidia on cotyledons to produce single cylindrical entities by this pathogen has been reported earlier by Gunasinghe et al. (2016d). Two different morphologies commonly observed for *N. capsellae* arthrospores were cylindrical (**Figures 9A, B** **)** or spherical (**Figures 9C, E** **)**. Initial observations are that the size of the arthrospores and the proportion of each morphology *in-vitro* vary depending on the growth conditions. This is not unusual as other dimorphic fungal species can produce a range of arthroconidia that differ in size and/or shape and the dominating type is determined by the substrate (King and Jong, 1976; Allermann et al., 1978; Kier et al., 1980; Barrera and Szaniszlo, 1985).

The development and release of arthroconidia appear to vary depending on the fungal species (Barrera and Szaniszlo, 1985). In both: *Mucor rouxii* and *Tricophyton mentagrophytes*, arthroconidia develop inside the hyphae as cylindrical conidial chains enveloped by the original hyphal cell wall but then change their morphology to spherical just before dehiscence (Takeo and Nishiura, 1974; King and Jong, 1976). Separation of the conidia could occur by autolysis of inter-septal material in the original hyphal wall that helps the adhesion of neighbouring septal walls of conidial chains or by dehiscence of the original hyphal wall (Barrera and Szaniszlo, 1985). The arthroconidia of *Antromycopsis brousonnetiae* developed a secondary cell wall inside the hyphae and the separation of conidia is by centripetal dehiscence of the original hyphal wall (Moore, 1977).

The frequently observed release mechanism for *N. capsellae* arthrospores produced *in-vitro* was dehiscence of original hyphae. Transverse dehiscence at the septa was more common (**Figures 9C–E**) than centripetal dehiscence (**Figure 9F**). However, the release of cylindrical arthrospores by autolysis of the original hyphal wall also occurred when arthrospores were produced from multi-celled macroconidia (**Figure 10**). There is evidence that arthrospores are a superior asexual spore type providing enhanced dissemination and better establishment on the host tissue by protecting cells from host lytic enzymes (Duran et al., 1973), increasing antibiotic production (Nash and Huber, 1971; Queener and Ellis, 1975), and by promoting surface adherence (Kurakado et al., 2020).

### Morphological Plasticity, Pathogen Biology, and Disease Epidemiology

Although white leaf spot has been a damaging disease on Brassicaceae, there is relatively little information on the pathogen’s biology and disease epidemiology (Gunasinghe et al., 2020). It has been suggested that the pathogen is under-reported as the causative agent, because of the difficulties in isolation related to the slow growth on artificial media (Gunasinghe et al., 2020). As demonstrated by this study the initial growth of *N. capsellae* on artificial media can initially mimic bacterial/yeast colonies (**Figures 2A, B** and **Figures 4C, D** **)**. This ability to form strikingly different colony morphology following *in-vitro* morphological transition creates difficulties in identification based on colony morphology which also potentially leads to under-reporting.

Morphological transformation in fungi plays critical roles in survival between seasons, dissemination, and host invasion and therefore, in virulence (Lin et al., 2015). Dimorphic or polymorphic fungi achieve this through the morphological transformation into single-celled spore types with different morphologies (blastospore or arthrospores) and/or varied physiologies (Odds and Kerridge, 1985) having altered tolerance to certain host physiological conditions making them more robust on hosts (Lin et al., 2015). In the absence of the teleomorph (sexual stage) *N. capsellae* infection cycle is not fully understood (Gunasinghe et al., 2020), as splash-dispersed multi-celled macroconidia are unlikely to be dispersed by wind (Fitt et al., 1992). The morphological transition on host leaves may provide a superior spore that can be efficiently disseminated through wind facilitating disease initiation and spread in the localities like North America, Asia, and Australia where the sexual stage and, therefore, ascospores are thought to be absent.

In the literature, polymorphism is defined as the ability of a fungus to take more than two different morphological forms (Mayer et al., 2013). *Candida albicans* provides a classic example for polymorphic pathogenic fungi, where it can form biofilms, different morphotypes for the single-celled blastospores (yeast phase), hyphae, and pseudo-hyphae (Odds and Kerridge, 1985; Kurakado et al., 2020). Formation of biofilm at the interface of yeast-to-hyphae transition was also observed for *N. capsellae* in this study. It is known that biofilm development and morphogenesis are interconnected phenomena controlled by the same set of genes for a given species (Lin et al., 2015; Kurakado et al., 2020). Further studies are recommended not only to answer the question of whether *N. capsellae* is dimorphic or polymorphic but also to define the decisive role/s of each morphotype produced understanding its morphogenesis.

## Conclusion

*N. capsellae* has the morphological plasticity to produce different morphologies in response to various environmental/external stimuli. During its morphogenesis, dikaryotic mycelium or asexually produced multi-celled macroconidia (2n) on leaf lesions in their parasitic phase initiate a single-celled yeast phase through the production of at least three types of morphologically distinguishable single-celled spore types. Multi-celled macroconidia on the host surface develop in three possible ways 1) they separate from the septa to produce multiple arthrospores that germinate as single entities, 2) they produce blastospores that increase in number rapidly by budding, and 3) they germinate as typical conidia by producing single or multiple germ tubes. This morphological plasticity of *N. capsellae* can be considered a cost-efficient strategy to provide a more resilient form of spores on the host enhancing inoculum potential and dissemination, both contributing to the pathogen’s virulence. A systematic study is recommended to fully understand *N. capsellae* multifaceted life cycle and to define the role of morphogenesis in relation to virulence, which is largely unknown.

## Data Availability Statement

The original contributions presented in the study are included in the article/supplementary material. Further inquiries can be directed to the corresponding author.

## Author Contributions

NG: Designed and conducted the work and drafted the manuscript. PD: Worked with NG to conduct the study and prepared the figures. SN, MB, and MY: Gave significant contribution to the conception and the design and reviewed the manuscript for critical and important intellectual content. All authors contributed to the article and approved the submitted version.

## Funding

Funding support for this research was provided by the School of Plant Biology at the University of Western Australia, and the Centre of Crop Health at the University of Southern Queensland.

## Conflict of Interest

The authors declare that the research was conducted in the absence of any commercial or financial relationships that could be construed as a potential conflict of interest.
